# Case report: congenital dislocation of the radial head –a two-in-one approach

**DOI:** 10.12688/f1000research.3-22.v1

**Published:** 2014-01-22

**Authors:** Raju Karuppal, Anwar Marthya, Rajendran V Raman, Sandhya Somasundaran

**Affiliations:** 1 Department of Orthopaedics, Government Medical College, Kozhikode, 673008, India; 2 Department of Orthopaedics, KMCT Medical College, Kozhikode, 673603, India

**Keywords:** Congenital dislocation of the radial head; Valgus deformity; Open reduction; Reconstruction; Two in one approach

## Abstract

**Background: **Congenital dislocation of the radial head of the elbow is rare. It is genetically transmitted in some cases and is often associated with syndromes, such as Nail-Patella syndrome, antecubital pterygium and ulnar dysplasia. About two thirds are posterior, with the remainder being either anterior (15%) or lateral (15%). The natural history of the condition is that symptoms are relatively benign, with only some limitation of motion and deformity. Treatment either involves early attempts at reconstruction or delayed intervention at skeletal maturity with radial head excision. We evaluated the radiographic and functional results of a two-in-one procedure (radial shortening and open reduction) in the treatment of congenital dislocation of the radial head of an eight year old girl.

**Objective: **To describe a technique for easy reduction and maintenance of normal radiocapitellar joint anatomy in cases of congenital dislocation of the radial head.

**Method: **We have introduced one modification to the Sachar’s method of open reduction by adding radial shortening. This can be described as a ‘two incision approach’ with the first incision for the radial shortening and the second for the open reduction of the radiocapitellar joint. The radial shaft was osteotomised first before we performed the radial head relocation. Then the overlapping part of radial shaft was trimmed. It was stabilized with a transarticular K wire fixation.

**Results: **At one year follow up, the elbow is stable with no valgus or fixed flexion deformity. Supination has increased to 40 degrees from zero degrees. An X-ray showed reformation of the radial head with good congruity of the radiocapitellar joint and correction of the radial bow.

**Conclusion: **As far as the authors are aware, this is the first report of congenital dislocation of the radial head being treated by radial shortening and open reduction of radiocapitellar joint through a two incision approach (two-in-one approach). This paper describes this new technique, which we implemented for easy reduction maintenance of normal radiocapitellar joint anatomy.

## Report of a new method of treatment

An eight year old, otherwise healthy girl, born out of non *consanguineous* marriage presented in 2012 with swelling of her left elbow joint, valgus deformity, limitation of movement, and occasional pain. The patient had no previous history of trauma or significant illness. Her elbow was apparently normal until the age of 4 years. At this point her mother noticed swelling and progressing deformity. On physical examination the radial head was dislocated posterolaterally and was not reducible. There was cubitus valgus (30 degrees) and 15 degrees of fixed flexion deformity. She had full flexion at her elbow but no supination of her forearm.

**Figure 1.**
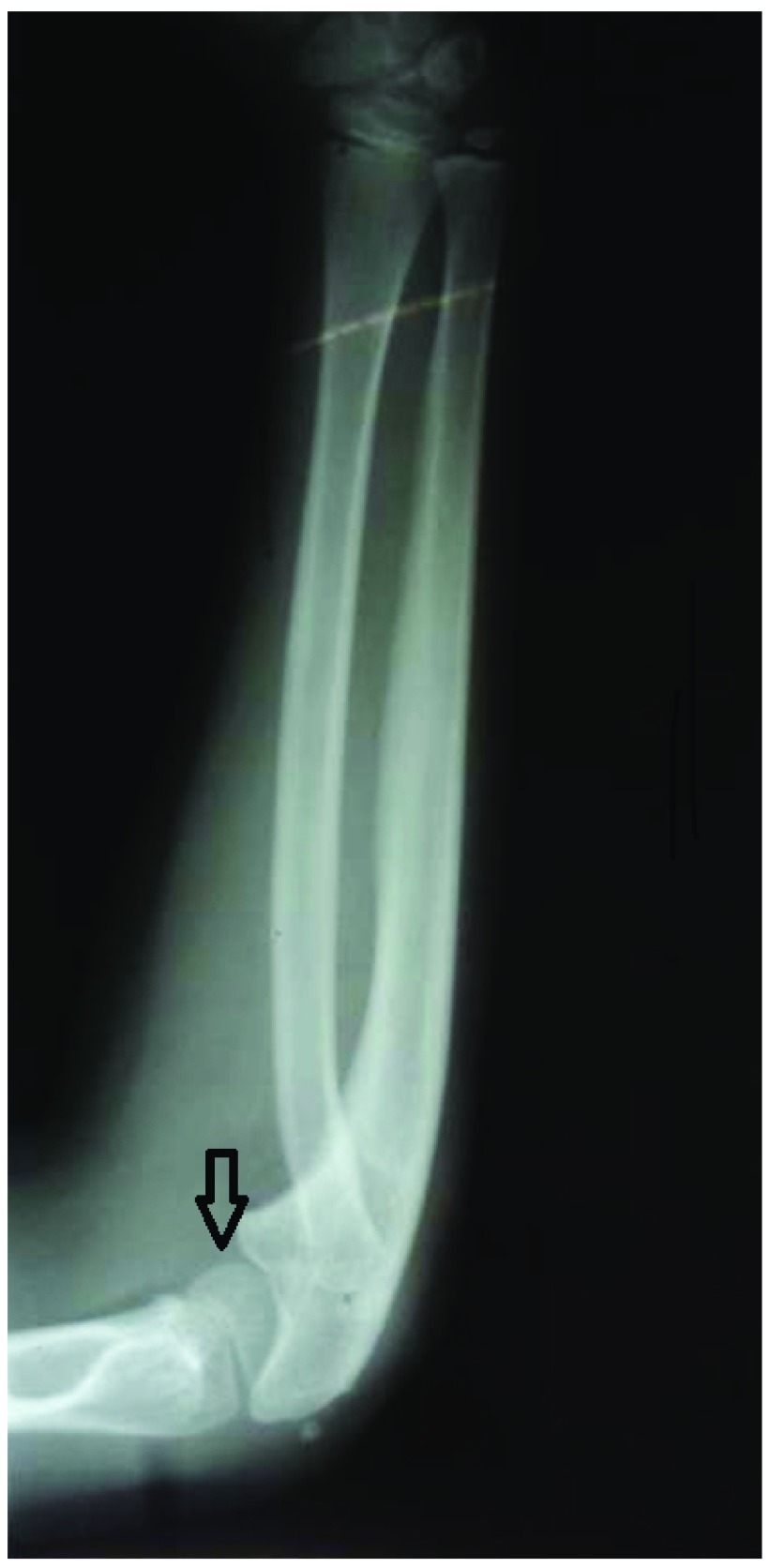
Pre-operative X-ray showing congenital radial head dislocation with the radius bowed anteriorly. The capitulum is hypoplastic.

**Figure 2.**
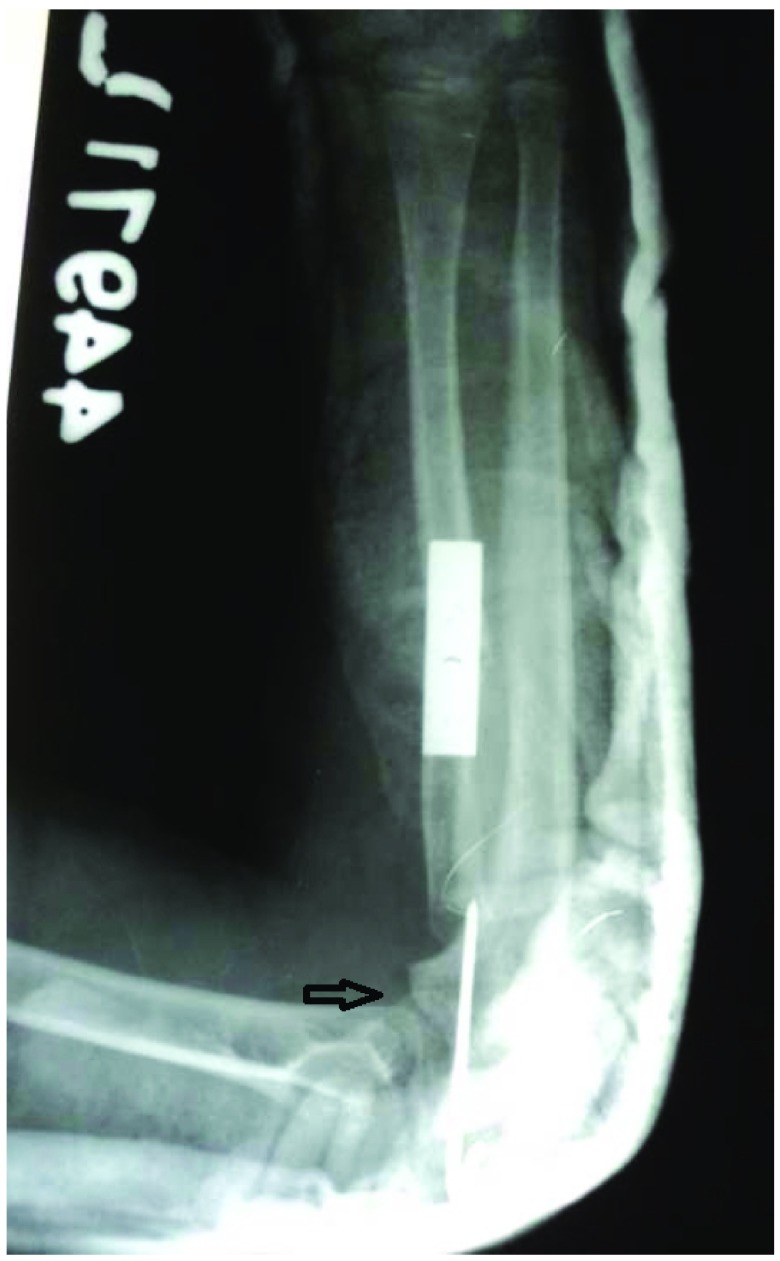
Immediate post-operative X-ray with radial shortening and fixation with plate and screws. The radial bow is corrected. The radial head is fixed with a transarticular pin.

An X-ray showed posterior dislocation of the radial head with a domed articular surface. McLaughlin’s line had not bisected the capitellum. The radius was bowed anteriorly and was relatively longer in relation to ulna. The capitulum was hypoplastic and flat. There was no evidence of any previous fracture ( Figure 1). An MRI showed posterior dislocation of the radial head and formation of a pseudo-joint with the adjacent margin of the ulna ( Figure 2). Due to relative radial lengthening and radial bow, it would have been difficult to reduce the radial head in the radiocapitellar joint. Hence we planned for reconstruction surgery by radial shortening and open reduction of the radiocapitellar joint through two incisions. Pre-operative templating was done to assess the amount of shortening required for the easy reduction and maintenance of adequate joint space.

## Operative technique

The novel two incision/two-in-one technique described here involves one modification to the Sachar’s method ^1^ of open reduction by adding a radial shortening step. Under tourniquet, we performed the surgery through a two incision approach. The first incision on the anterior aspect of the proximal forearm was for radial shortening (Henry approach) and the second incision on the lateral part of the elbow was for open reduction of the radiocapitellar joint (Kocher’s approach). The proximal radial shaft was osteotomised first before we performed the radial head relocation to facilitate the reduction, which reduces the risk of osteonecrosis ^2^. Then the overlapping part of radial shaft was trimmed with a bone nibbler, similar to the procedure of femoral shortening in the treatment of a dysplastic dislocated hip joint ^3^. The radial shaft was stabilized by a transradiocapitellar fixation with a 1.8 mm K wire with the elbow in flexed position.

Post-operatively, the elbow was immobilized with an above elbow plaster splint, in 90° of flexion, for six weeks at which point the K-wire was removed and the elbow was mobilized. The patient was further managed with controlled elbow movement exercises and physiotherapy for the next three months to improve the range of movement and muscle strength.

**Figure 3.**
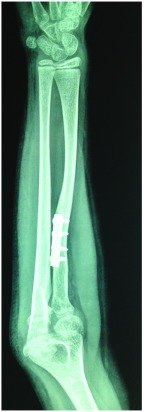
One year follow up showing remodeling of the radial head.

At one year follow up in 2013, the patient’s elbow was stable with no valgus or fixed flexion deformity. Supination had increased to 40 degrees from zero degrees. An X-ray showed reforming of the radial head articular surface with good congruity of the radiocapitellar joint with no deformity of the radial shaft ( Figure 3).

## Discussion

Congenital dislocation of the radial head is the most common congenital elbow abnormality ^1^ and usually occurs in association with other conditions (60% of the time), but can also occur in isolation ^4^. The more common associated conditions include lower extremity anomalies, scoliosis, mental retardation, and nail-patella and Klippel-Feil syndromes ^5^. The condition is usually bilateral, but some unilateral cases have been described ^5, 6^. When unaccompanied by other radial or systemic conditions, it is almost always bilateral. The majority of radial head dislocations are posterior (65% of cases), followed by anterior (~15%) and lateral (~15%) ^7^. It is often not noted until the age of four or five at which time some limitation of motion or deformity becomes evident ^7^. Our case is an isolated unilateral congenital dislocation of the radial head noted at the age of four years.

In our case, we anticipated difficulty in reducing the radial head to the radiocapitellar joint, due to relative radial lengthening and radial bow. Hence we planned for reconstruction surgery by radial shortening and open reduction of the radiocapitellar joint through two incisions. Pre-operative templating was done to assess the amount of shortening required for the easy reduction and maintenance of adequate joint space.

The dislocation of radial head and its associated features are now believed to be triggered by failure of development of a normal capitulum, which deprives the developing radial head of the contact pressure required for normal development and results in malformation of the radiocapitellar joint ^8^. The symptoms are relatively benign, with only some limitation of motion and deformity ^9^. Early radiographic findings are subtle due to the absence of the capitulum and radial head ossification centers ^9^. Before radial head ossification (~ five years) a line drawn along the shaft of the radius should normally bisect the capitellum ossification center (McLaughlin’s line) but did not in this case ^10^.

McFarland (1936) ^11^ described the radiological signs which he believed distinguished the congenital from the traumatic in unilateral dislocations. However the convex radial head, flattening of the capitellum and anterior angulation of the ulna are regarded as characteristic of congenital dislocation are clearly seen as a result of the injury as well ^12^.

Generally, patients become symptomatic by adolescence and are treated by radial head resection. Surgery at an earlier age with open reduction and ligament reconstruction may offer advantages over late radial head resection ^1^. Early reconstruction may prevent the long term complication of pain, loss of motion, deformities and osteochondral loose bodies ^13^. Ideally the care of congenital dislocation of the radial head would involve open reduction and restoration of normal anatomy. The logic is that if the radial head can be reduced early, the deformity of the capitellum and the forearm may not occur or remodel with growth ^13^.

We agree with De Boeck ^14^ that reconstruction of the annular ligament seems unnecessary, and that unreduced radial head dislocations, therefore, may be treated by simple open reduction and fixation for six weeks with a transarticular pin. This is why the annular ligament was not reconstructed in our case. Although much of the literature refers to the treatment of radial head dislocation by means of an ulnar osteotomy, as described by Hiramaya and coworkers ^15^, the main indication of this procedure is the presence of residual deformity of the ulna or radius with a concave radial head articular surface ^16^. In our case there was no deformity of the ulna, but there was lengthening of the radius with a domed radial head.

We were cautious not to excise the radial head in our case because of the risk of secondary subluxation of the distal radio-ulnar joint due to proximal migration of the radius ^17^. We believe on the basis of our results that surgical correction is fully justified in irreducible dislocation of the radial head. We also believe that shortening of the radius would have been necessary to achieve reduction in cases where there is radial lengthening.

## Conclusion

No reported case of congenital dislocation of the radial head treated by radial shortening and open reduction of the radiocapitellar joint through two incision approaches (two-in-one approach) has hitherto been available, as far as the authors are aware. This paper describes such a case, where we used this technique for the easy reduction of the radiocapitellar joint in order to maintain normal joint anatomy.

## Consent

Consent was obtained from the patient and her mother for the novel technique to be conducted. We also obtained the consent from them for use of their information and images for publication in this article.
