# Supplementary material for: Case report: congenital dislocation of the radial head –a two-in-one approach
Source: F1000Res. 2014 Jan 22;3:22. [Version 1] doi: 10.12688/f1000research.3-22.v1 (PMC4857746; doi:10.12688/f1000research.3-22.v1)
Supplement: Peer review history

# Peer review: Referee response for version 1

**DOI:** 10.5256/f1000research.3476.r13345

**Published:** 2016-05-04

**Authors:** Sandeep Vijayan

**Affiliations:** 1 Department of Orthopedics, Kasturba Medical College, Manipal University, Manipal, Karnataka, India

**Positive points:**

The article is well designed and authored mentioning the surgical management of a rare congenital entity. The case report and surgical steps are clearly described. As per the report, early reduction of the radial head helps in better remodeling of the radio-capitellar joint similar to early reduction of hip in developmental dysplasia of the hip.

**Some of my queries and suggestions are as follows:**

1. Even though, authors are unaware of radial shortening and open reduction of the radiocapitellar joint through two incisions to the best of their knowledge, I wish to draw their attention to the paper by Hui Taek Kim in Journal of Paediatric Orthopaedics. On 3 elbows with congenital dislocation of radial head (2 patients) they did open reduction of radial head through a posterior approach and radial shortening through a midshaft approach.
2. As the natural history of congenital dislocation of the radial head is benign, the readers would be interested and benefited to know what are / were the indications for surgical intervention in these / this case. Authors could discuss briefly about situations where surgical management is not needed.
3. Even though, authors have mentioned about MRI findings (figure 2), the article does not carry any MRI picture. This minor mistake might be rectified and the role of MRI, if any, in the management of congenital dislocation of the radial head could be discussed in brief.
4. Anatomic relationship between ulnohumeral, radio-capitellar and proximal radio-ulnar joint must be maintained in a skeletally immature patient for normal development to occur. It would be worthwhile if authors can mention about the status of ulno-humeral and proximal radio-ulnar joints in the pre and post operative radiographs.
5. Readers would be interested to know if any rotation of the proximal fragment was done to improve supination before transfixation with K wire and plate fixation.
6. It would be worth mentioning in the ‘operative technique’ about fixation of the osteotomy site with plate and screws.
7. What was the position of the forearm in the cast postoperatively?
8. Cadaveric studies have shown that the annular ligament is the most important ligament in stabilizing the radial head and the proximal radio-ulnar joint during all phases of rotation while the interosseous membrane contribute significantly only during the terminal phase of supination. So the claim made by authors that annular ligament reconstruction is not needed warrants further justification.
9. The main problem in congenital dislocation of the radial head is the hypoplasia of capitullum and the mismatch between the dome shaped radial head and capitellum and alteration in the radial neck-head angle leading to re-dislocation/subluxation. It would be more informative if the authors could provide a lateral view of the elbow at final follow up to show the maintenance of the reduction and remodeling of the radio-capitellar joint.
10. At one year follow up, the radiograph shows a positive ulnar variance. Authors could mention about the function of the wrist as well.
11. It would be worth mentioning that a longer follow up is required to know the final outcome of the surgery as the child is skeletally immature.

I have read this submission. I believe that I have an appropriate level of expertise to confirm that it is of an acceptable scientific standard.

# Peer review: Referee response for version 1

**DOI:** 10.5256/f1000research.3476.r10118

**Published:** 2015-09-03

**Authors:** Kishore Puthezhath Menon

**Affiliations:** 1 Department of Orthopedics, Amala Institute of Medical Sciences, Thrissur, Kerala, India

Apart from the rarity, the case report attempts to convey that the treatment options available for congenital dislocation of radial head are either early reconstruction or late radial head excision and the former is ideal. 

But the fact of the matter is that, this is not in alignment with established evidence. "A congenitally dislocated radial head is irreducible manually or surgically because of adaptive changes in the soft tissues and the absence of normal surfaces for articulation with the ulna and humerus. Consequently, open reduction of the dislocation and reconstruction of the annular ligament in childhood are not advised. Any impairment of function usually is caused by restriction of rotation of the forearm; in children, physical therapy to improve this motion is the only treatment indicated. If pain persists into adulthood, the radial head and neck can be excised." Campbell's Operative Orthopaedics, 12e.

Though not recommended by standard textbooks, if a reconstruction is planned as in the present report, annular ligament reconstruction is necessary as per available literature. De-Boeck's recommendations against this in case of chronic acquired isolated radial head dislocations do not seem to be applicable for congenital dislocations. Further more, as per Sachar, reconstruction is better than radial head excision in adulthood only if it is performed before 2 years.

Figure 2 is not a MRI picture. Do MRIs have any role in determining whether a rotation osteotomy is to be done or not? How can we measure cubitus valgus angle in patients with fixed flexion deformity? A detailed explanation on method of pre operative templating may help curious surgeons.

I have read this submission. I believe that I have an appropriate level of expertise to confirm that it is of an acceptable scientific standard, however I have significant reservations, as outlined above.

# Peer review: Referee response for version 1

**DOI:** 10.5256/f1000research.3476.r5769

**Published:** 2014-08-13

**Authors:** Nikolaos Gougoulias

**Affiliations:** 1 Trauma & Orthopaedics Department, Frimley Park Hospital, Frimley, Surrey, UK

This is a nicely presented case report of the relatively uncommon condition of congenital elbow dislocation. The authors describe the rationale of management and the surgical technique with clarity. They also provided evidence of follow up. The discussion is nicely written, providing information regarding the background of the condition they treated, putting the reader, who is not a specialist in the field, in the correct context. 

I approve the article's indexation. Its current version could be further improved if the authors provided clinical pictures of the patient's forearm postoperatively in different positions, indicating for example the ability to perform supination/pronation movements (if such photos were available).

I have read this submission. I believe that I have an appropriate level of expertise to confirm that it is of an acceptable scientific standard.
